# Gastrointestinal Stability and Microbiota-Mediated Deglycosylation of Triterpene Glycosides from *Cimicifuga racemosa* Extract Ze 450

**DOI:** 10.3390/molecules31213928

**Published:** 2026-11-01

**Authors:** Roman Senn, Benoit Pugin, Verena M. Merk, Denisa Mujezinovic, Janina Zünd, Georg Boonen, Christophe Lacroix, Veronika Butterweck

**Affiliations:** 1 Medical Department, Max Zeller Söhne Aktiengesellschaft, 8590 Romanshorn, Switzerland; roman.senn@zellerag.ch (R.S.); verena.merk@zellerag.ch (V.M.M.); georg.boonen@zellerag.ch (G.B.); 2 Laboratory of Food Biotechnology, Eidgenössische Technische Hochschule Zürich, 8092 Zürich, Switzerland; benpugin@hotmail.com (B.P.); denisa.m@outlook.com (D.M.); zuendjanina@gmail.com (J.Z.); christophe.lacroix@hest.ethz.ch (C.L.)

**Keywords:** *Cimicifuga racemosa*, Ze 450, menopause, menopausal symptoms, microbiome, actein, 23-epi-26-deoxyactein, triterpene glycosides, biotransformation, aglycone, gut microbiota

## Abstract

Triterpene glycosides (TTGs) are characteristic constituents of *Cimicifuga racemosa* extract Ze 450 and may contribute to its pharmacological effects in the treatment of menopausal symptoms. This exploratory study investigated their gastrointestinal stability, transformation during incubation with human fecal microbiota, and potential effects on microbial community composition and short-chain fatty acid (SCFA) production. The stability of 23-epi-26-deoxyactein was assessed during simulated oral and gastric digestion using the INFOGEST protocol. Microbiota-associated biotransformation of actein, 23-epi-26-deoxyactein, cimigenol-3-β-D-xyloside, and cimigenol-3-α-L-arabinoside was subsequently examined during 48 h anaerobic ex vivo cultivation with fecal microbiota from eight female donors. 23-Epi-26-deoxyactein remained largely stable during simulated oral and gastric digestion. During fecal incubation, all investigated TTGs were depleted and the corresponding aglycones were detected. Quantification of the 23-epi-26-deoxyactein aglycone revealed marked donor-dependent differences, indicating substantial interindividual variability in TTG biotransformation. Individual TTGs had little effect on microbial community composition or SCFA profiles, whereas Ze 450 induced a small but significant shift in overall community structure without substantially affecting SCFA production. These findings demonstrate extensive microbiota-mediated TTG deglycosylation and highlight marked interindividual variability in TTG metabolism, providing a rationale for further investigation of microbiome-dependent differences in the pharmacological fate of Ze 450.

## 1. Introduction

Women spend approximately one-third of their lives in the postmenopausal state, and many experience vasomotor symptoms, sleep disturbances, anxiety, mood changes, and urogenital complaints that substantially impair quality of life [1,2]. Although menopausal hormone therapy remains the most effective treatment for many women, contraindications and safety concerns have increased the demand for evidence-based non-hormonal therapeutic alternatives [3].

One such alternative is Ze 450, an ethanolic extract of *Cimicifuga racemosa* L. Nutt. (black cohosh, Ranunculaceae), which is approved as an herbal medicinal product in 28 countries for the treatment of menopausal symptoms, including hot flushes, sweating, sleep disturbances, nervousness, and mood swings [4]. Multiple clinical studies have demonstrated its efficacy together with a favorable safety profile [5,6,7,8]. Despite its established clinical use, the molecular mechanisms underlying its therapeutic effects are not yet fully understood.

Unlike menopausal hormone therapy, Ze 450 does not exert its pharmacological activity through classical estrogen receptor activation [9,10]. Instead, accumulating evidence suggests that Ze 450 acts as a metabolic modulator by activating AMP-activated protein kinase (AMPK), thereby inducing systemic metabolic adaptations associated with improved cellular energy homeostasis and reduced inflammatory signaling [11,12,13,14,15,16,17,18]. Whether AMPK activation is causal for these effects or primarily reports the underlying change in cellular energy state has not been established. More recently, experimental studies have demonstrated additional metabolic effects of Ze 450, including reductions in visceral adiposity and improvements in metabolic parameters in preclinical models, suggesting a broader pharmacological profile beyond symptom relief [16,19]. As with most herbal medicinal products, the total extract is regarded as the active pharmaceutical ingredient, whereas the contribution of individual constituents remains incompletely understood. Among these constituents, cycloartane-type triterpene glycosides (TTGs), particularly actein and 23-epi-26-deoxyactein, have recently attracted considerable attention after being identified as major contributors to the AMPK-activating properties of Ze 450 using machine learning-assisted analyses [3,20].

Saponins as a compound class are frequently assumed to be poorly absorbed because of their high molecular mass and polarity [21]. For the cycloartane glycosides of *Cimicifuga*, however, compound-specific data indicate partial rather than negligible absorption: ab-solute oral bioavailability in rats was 27 to 49% for 23-epi-26-deoxyactein and 33 to 48% for 25-O-acetylcimigenoside, whereas cimicifugoside H-1 was poorly absorbed (2 to 7%) [22], and intact 23-epi-26-deoxyactein is detectable in human serum after oral administration of a standardised extract [23]. The fraction that escapes absorption in the upper gastrointestinal tract reaches the colon, where microbial deglycosylation may generate metabolites with distinct physicochemical and pharmacological properties. This process should therefore be viewed as an additional metabolic pathway rather than a prerequisite for absorption in the upper intestine. Whether the resulting aglycones are pharmacologically active or absorbed from the colon remains unknown. The human gut microbiota represents a highly diverse and metabolically active microbial ecosystem that contributes to numerous aspects of host physiology and generates a wide range of bioactive metabolites [24,25]. Increasing evidence indicates that the gut microbiota plays a pivotal role in the biotransformation of phytochemicals, thereby influencing their bioavailability, biological activity, and ultimately therapeutic efficacy [26]. Conversely, phytochemicals themselves may alter microbial composition and function, highlighting the bidirectional nature of host–microbiome interactions and their growing importance in phytopharmacology [27]. Importantly, the composition and metabolic capacity of the gut microbiota vary considerably between individuals, potentially leading to substantial differences in the metabolism and activity of herbal medicines [28,29]. Moreover, menopause itself has been associated with alterations in gut microbial composition and diversity, which may further influence these processes [30,31].

Despite the widespread clinical use of Ze 450, little is known about its interaction with the human gut microbiota. In particular, it remains unclear whether its characteristic triterpene glycosides undergo microbiota-mediated biotransformation, whether such metabolism differs between individuals, and whether Ze 450 itself influences microbial community composition or metabolic activity.

Therefore, the present study investigated the reciprocal interaction between Ze 450 and the human gut microbiota by assessing the stability of the major triterpene glycosides under simulated gastrointestinal conditions, their microbiota-mediated biotransformation using ex vivo human fecal fermentations, and the effects of Ze 450 and its TTGs on microbial community structure and function. By addressing these questions, this study provides new insights into the role of the gut microbiome in the pharmacology of Ze 450 and establishes a foundation for future investigations into microbiome-dependent variability in therapeutic response.

## 2. Results

### 2.1. Stability of the Triterpene Glycosides Under Simulated Oral and Gastric Conditions

Prior to the investigation of the effects of selected triterpene glycosides (TTGs) (Figure 1) and Ze 450 on the gut microbiota, their stability during the oral and gastric phaseswas assessed using the INFOGEST in vitro digestion model [32]. Recovery of TTGs was monitored using 23-epi-26-deoxyactein, one of the most abundant and characteristic cycloartane triterpene glycosides of Cimicifuga racemosa and an established analytical marker for black cohosh extracts, during the oral and gastric phases [33]. No substantial degradation was observed under these conditions (Figure S1), suggesting that TTGs may reach the colon with minimal degradation. Therefore, the administered TTGs and Ze 450 suspension were considered representative of the compounds encountered by the gut microbiota.

### 2.2. Effects of TTGs and Cimicifuga Extract Ze 450 on Gut Microbiota Composition

The effects of individual *Cimicifuga racemosa* triterpene glycosides (TTGs) (30 µg/mL) and the entire Ze 450 extract (300 µg/mL) on gut microbiota composition were assessed after 48 h of anaerobic cultivation of fecal samples from eight human donors. The concentration of 30 µg/mL used for the individual TTGs was based on the experimental conditions described by Chauveau et al. [34], who derived the selected concentration by considering oral exposure, phytochemical content, gastrointestinal stability and absorption, as well as colonic volume and transit time. Microbial communities were incubated with actein (TTG-1), 23-epi-26-deoxyactein (TTG-2), cimigenol-3-β-D-xyloside (TTG-3), cimigenol-3-α-L-arabinoside (TTG-4), or the complete Ze 450 extract. Neither the number of observed amplicon sequence variants (ASVs) nor the Shannon diversity index differed significantly from the DMSO control (Figure 2A), indicating that microbial richness and diversity were largely maintained across treatments. A decrease in Shannon diversity was observed in only one donor (Donor 2) following treatment with the complete Ze 450 extract (Figure 2A). Bray–Curtis β-diversity analysis revealed only minor treatment-induced changes in community composition (Figure 2B). Samples treated with individual TTGs clustered closely with the DMSO controls (Bray–Curtis distance = 0 if identical to controls), whereas the complete Ze 450 extract induced the largest, albeit modest, compositional shift (maximum Bray–Curtis distance: 0.31 ± 0.05; Figure 2B). Community composition following treatment with the complete Ze 450 extract differed significantly from that observed with each individual TTG (adjusted *p* = 0.02 for all comparisons). Overall, individual TTGs did not measurably alter gut microbiota composition relative to the control, whereas the complete Ze 450 extract induced a small but significant shift in overall community structure.

Baseline taxonomic profiling highlighted substantial inter-individual variation across donor microbiotas (Figure 2C). Although *Lachnospiraceae* predominated across all donors, donors 1 and 2 exhibited *Prevotellaceae*-enriched communities, whereas donors 3–8 were dominated by *Bacteroidaceae*. In agreement with the limited shifts observed in β-diversity, individual TTGs had little impact on family-level community composition (Figure 2C and Figure 3). Similarly, the complete Ze 450 extract induced only modest changes in the relative abundance of microbial families. Although not statistically significant, a reduction in *Streptococcaceae* abundance was observed following treatment with Ze 450 (Figure 3A). At the genus level, Ze 450 significantly increased the relative abundance of *Faecalibacterium* (*p* = 0.033) and decreased that of *Lachnospiraceae* UCG-004 (*p* = 0.047) (Figure 3B).

### 2.3. Effects of TTGs and Cimicifuga Extract Ze 450 on Microbial Metabolic Profiles

Short-chain fatty acids (SCFAs) are organic metabolites produced by the gut microbiota through the fermentation of dietary fiber [32]. They play important roles in both local intestinal and systemic physiological processes by regulating immune responses, cellular signaling, and host metabolism [32]. To evaluate the effects of TTGs and the *Cimicifuga racemosa* extract Ze 450 on SCFA production, supernatants from fecal cultures of the eight donors grown in the presence of the respective treatments were analyzed. Consistent with the limited changes observed in microbial community composition, neither the individual TTGs nor Ze 450 substantially affected microbial carbohydrate metabolism. Relative concentrations of acetate, propionate, butyrate, formate, and succinate remained similar to those measured in control cultures (Figure 4).

### 2.4. Aglycone Recovery Appears to Be Donor- and Thus Microbiome-Specific

We next investigated whether inter-individual differences in microbial community composition influenced TTG metabolism. To assess microbiota-mediated TTG biotransformation, the conversion of 23-epi-26-deoxyactein (TTG-2) into its corresponding aglycone, 27-deoxyacetylacteol, was analyzed following incubation of TTG-2 with fecal microbiotas from individual donors. LC–HRMS analysis revealed depletion of the parent compound together with the formation of 27-deoxyacetylacteol, demonstrating microbiota-mediated hydrolysis of the glycosidic moiety (Figure 5A). Similar substrate depletion and aglycone formation were also observed for TTG-1, TTG-3, and TTG-4 (Figures S2–S4), indicating that all investigated TTGs were susceptible to microbial deglycosylation.

Quantification of 27-deoxyacetylacteol by UHPLC-qTOF-MS revealed substantial inter-donor variability in aglycone recovery following TTG-2 incubation (Figure 5B). Recovery rates ranged from approximately 10% to 70%, depending on the donor microbiota. The highest recovery was observed for donors 5–7, whereas donors 1, 3, 4 and 8 showed intermediate recovery and donor 2 exhibited the lowest recovery. These findings demonstrate marked donor-dependent differences in TTG biotransformation efficiency and suggest that microbiota composition influences the extent of aglycone formation.

## 3. Discussion

The present study demonstrates that major cycloartane triterpene glycosides (TTGs) of *Cimicifuga racemosa* extract Ze 450 are extensively metabolized by the human gut microbiota. Under the experimental conditions and at the concentrations tested, neither the individual TTGs nor Ze 450 substantially altered microbial community composition or short-chain fatty acid (SCFA) production. The extract concentration used in the present study should be regarded as a defined challenge concentration rather than as a direct estimate of physiological colonic exposure. A first approximation is nevertheless possible: a 6.5 mg tablet of Ze 450 contains approximately 0.76 mg of total triterpene glycosides, expressed as 23-epi-26-deoxyactein, corresponding to approximately 10% of the extract. Assuming distribution of this total amount in a proximal colonic volume of approximately 200 mL gives a theoretical concentration of approximately 3.8 µg/mL, i.e., about 4 µg/mL total TTG. By comparison, the experimental Ze 450 concentration of 300 µg/mL corresponds to approximately 35 µg/mL total TTG equivalents, or roughly ninefold above this theoretical estimate. Likewise, the 30 µg/mL concentration used for each individual TTG exceeds the estimated physiological concentration of any single TTG by an even greater margin. The concentrations used in the present study should therefore be regarded as defined experimental challenge concentrations rather than as estimates of physiological colonic exposure. All investigated TTGs disappeared during anaerobic incubation, accompanied by the formation of corresponding aglycones. However, aglycone recovery differed markedly between donors, indicating pronounced interindividual variability in microbial TTG metabolism. Previous ex vivo studies have demonstrated that the human gut microbiota can extensively transform constituents of herbal medicinal products. Chauveau et al. [34] investigated pharmacologically active compounds from valerian and St. John’s wort using short-term fecal microbiota fermentations, while Thumann et al. [35] examined the microbial biotransformation of constituents of the multi-herbal medicinal product STW 5. Similar to the present findings, these studies showed substantial microbiota-mediated transformation of plant-derived constituents, while overall microbial community structure was only minimally affected. The present study extends these observations to the characteristic cycloartane triterpene glycosides of *Cimicifuga racemosa*. In addition to demonstrating formation of the corresponding aglycones, quantitative analysis of 23-epi-26-deoxyactein revealed pronounced donor-dependent differences in biotransformation, suggesting substantial interindividual variability in microbial metabolic capacity. To our knowledge, the microbiota-mediated transformation of these characteristic *Cimicifuga* TTGs has not previously been systematically investigated using human fecal microbiota.

Our findings may also be relevant in light of previous pharmacokinetic observations for 23-epi-26-deoxyactein in humans [23]. Following oral administration of a standardized black cohosh extract, 23-epi-26-deoxyactein was detectable in serum and showed dose-proportional systemic exposure, whereas less than 0.01% of the administered dose was recovered unchanged in urine within 24 h. No major phase I or phase II metabolites were detected in clinical samples or following incubation with human hepatocytes [23]. Although these findings do not establish a role of the gut microbiota, the low recovery of unchanged compound and the proposed contribution of biliary and gastrointestinal elimination are consistent with the possibility that intestinal microbial transformation contributes to the overall metabolic fate of 23-epi-26-deoxyactein [11,12,13,14,17,23,36].

The gut microbiome is increasingly recognized as an important determinant of xenobiotic metabolism, bioavailability, and therapeutic response [37,38]. Microbial enzymes can convert poorly absorbed glycosylated plant constituents into metabolites with altered physicochemical and pharmacological properties [39]. In the present study, marked donor-dependent differences in TTG biotransformation were observed, indicating substantial interindividual variability in microbial metabolic capacity. Although the fecal donors in the present study were healthy women aged 21–37 years, these findings may be of particular interest in the context of menopause. Menopause has been associated with changes in gut microbial composition and function [30,40], including alterations in microbial pathways involved in hormone metabolism. It is therefore conceivable that menopause-related differences in microbial functional capacity could also influence the biotransformation of plant-derived compounds such as *Cimicifuga* triterpene glycosides. This possibility remains speculative, however, and cannot be addressed by the present study. Dedicated studies in peri- and postmenopausal women will be required to determine whether TTG metabolism differs in these populations [30,40].

Given the bidirectional relationship between sex hormones and the gut microbiome [41], including microbial deconjugation of steroid hormones, functional changes in the menopausal microbiome may be relevant beyond hormone metabolism alone [30,31,41]. Thus, interindividual differences in microbial enzyme activity may influence the metabolic fate of biologically active compounds independently of broad taxonomic composition.

The pronounced donor-dependent differences observed in the present study provide a rationale for investigating whether microbial metabolic capacity contributes to variability in the pharmacological fate of *Cimicifuga racemosa* constituents. Similar microbial producer and non-producer phenotypes have been described for other plant-derived compounds, including soy isoflavones in peri- and postmenopausal women [42]. The high-, intermediate-, and low-recovery phenotypes observed here may represent analogous differences in microbial metabolic capacity, although this exploratory classification requires confirmation in substantially larger cohorts and does not permit conclusions regarding clinical treatment response.

Despite considerable differences in baseline microbiome composition between donors, incubation with individual TTGs or Ze 450 resulted in only minor taxonomic shifts. Similarly, SCFA profiles remained largely stable, suggesting preservation of core microbial fermentation activity. Together, these findings indicate that the tested concentrations of TTGs and Ze 450 did not substantially disrupt microbial community structure or major metabolic outputs during short-term incubation. This treatment-associated stability should be distinguished from baseline microbiome differences associated with menopausal status, which vary across studies and populations [30,40]. The absence of substantial treatment-associated changes therefore does not imply that menopausal status has no influence on the microbiome, but rather that TTGs and Ze 450 did not markedly perturb the established donor-specific communities under the investigated conditions. The distinction between microbial composition and function is also relevant, as functional alterations may occur without detectable changes in overall diversity or taxonomy [30,40].

In contrast to the limited effects on microbial community structure, TTG metabolism showed substantial donor-dependent variability. Based on quantitative recovery of 27-deoxyacetylacteol, donors 5–7 showed the highest recovery (approximately 60–70%), donors 1, 3, 4, and 8 intermediate recovery (approximately 30–40%), and donor 2 the lowest recovery (approximately 10%). Although these groups are exploratory, they illustrate marked interindividual differences in the capacity of fecal microbiota to generate detectable aglycones. Such donor-specific metabolic phenotypes are consistent with the growing recognition that microbial metabolism of dietary phytochemicals is determined primarily by the functional enzymatic repertoire of the intestinal microbiota rather than by its overall taxonomic composition [41].

Qualitative analysis demonstrated the disappearance of all investigated TTGs, including arabinosides and xylosides, accompanied by formation of the corresponding aglycones, indicating that microbial deglycosylation represents an important initial step in TTG metabolism. However, quantitative aglycone recovery did not fully account for disappearance of the parent glycosides and differed between TTGs and donors. The resulting aglycones may therefore undergo further microbial transformation, including fragmentation, reduction, oxidation, deacetylation, or hydroxylation. This is consistent with sequential microbial phytochemical metabolism, in which initial deglycosylation is followed by additional biotransformation steps generating structurally diverse metabolites [43,44]. Consequently, unidentified downstream metabolites may contribute to the biological effects of *Cimicifuga racemosa* extracts and warrant further characterization.

Microbiome composition did not reveal a simple relationship between major bacterial families and aglycone recovery. *Lachnospiraceae* were prevalent across donors, and high-recovery donors also showed relatively high abundances of *Bacteroidaceae* and *Ruminococcaceae*; donor 6 additionally exhibited comparatively high *Sutterellaceae* abundance. However, similar dominant families occurred in donors with lower recovery, indicating that family-level abundance alone does not explain TTG metabolism. This is consistent with microbial functional redundancy and with evidence that phytochemical-metabolizing capacity may differ substantially at species- and strain-level resolution [41].

The occurrence of *Prevotellaceae* and *Sutterellaceae* is of interest because menopause-associated differences involving related taxa have been reported [30,31]. However, such associations should not be interpreted as evidence of a direct role in TTG metabolism. Donor 2, for example, showed the lowest 27-deoxyacetylacteol recovery despite a high abundance of *Lachnospiraceae* and additionally exhibited comparatively high *Prevotellaceae* abundance. Given the single-donor observation and the metabolic heterogeneity within bacterial families and genera, attribution of this phenotype to *Prevotellaceae* or any other individual taxon would be premature. Likewise, the intermediate-recovery donors showed heterogeneous microbial profiles despite comparable metabolic phenotypes. Together, these findings suggest that, at the taxonomic resolution available in the present study, TTG metabolism is better described by microbial metabolic phenotype than by the relative abundance of individual bacterial families or genera. This does not exclude the involvement of specific species, strains, or microbial enzymes in TTG biotransformation. Rather, it highlights the need for functional approaches capable of resolving these determinants.

This interpretation is consistent with studies distinguishing taxonomic composition from microbial functional potential. Peters et al. demonstrated menopause-associated differences in functional pathways and hormone-deconjugating orthologs, whereas Liu et al. found that predicted microbial metabolic pathways differentiated women with menopausal syndrome more clearly than overall microbial diversity [30,40]. Functional characterization may therefore be more informative for understanding TTG metabolism than broad taxonomic composition alone. Future studies combining shotgun metagenomics with metabolomics and targeted enzyme analyses, followed by experiments with isolated strains or defined microbial consortia, could identify the genes, enzymes, and microorganisms responsible for TTG biotransformation.

The applied concentration should also be considered when interpreting the findings. The TTG concentration of 30 µg/mL was selected according to the experimental approach of Chauveau et al. [34], who justified this concentration based on oral dose, constituent content, gastrointestinal stability, limited absorption, colonic volume, and transit time. As outlined above, the expected total luminal TTG concentration at the therapeutic dose lies in the single-digit µg/mL range. The concentration used here should therefore be regarded as a defined experimental challenge concentration rather than as an established in vivo colonic exposure. Future studies should evaluate concentration-dependent microbial metabolism across a broader concentration range, including exposure levels more closely reflecting estimated physiological colonic concentrations.

Several limitations should be acknowledged. First, only eight female donors were included. No formal sample-size calculation was performed because the study was exploratory and no prior data were available to support a meaningful effect-size estimate. Given the small number of donors, the observed donor-dependent differences should be considered exploratory and cannot be used to estimate the prevalence or distribution of such metabolic phenotypes in the wider population. Confirmation in substantially larger donor cohorts will therefore be required. Although this design revealed substantial interindividual variability, it is insufficient to establish robust metabolic subgroups or reliable associations between individual taxa and TTG metabolism. The proposed recovery phenotypes are therefore hypothesis-generating and require confirmation in larger, well-characterized cohorts accounting for menopausal status, age, hormonal exposure, diet, ethnicity, medication use, and metabolic health [30,31,40]. Second, the short-term ex vivo batch fermentation model cannot fully reproduce the human colon, including host–microbiome interactions, absorption processes, repeated exposure, and the hormonal environment. Third, 16S rRNA gene sequencing provides limited species-, strain-, and functional resolution and therefore cannot identify the microorganisms or enzymes responsible for TTG metabolism. Finally, only selected parent TTGs and aglycones were quantified, and incomplete aglycone recovery indicates the formation of additional unidentified metabolites. Continuous fermentation models, larger donor cohorts, integrated functional microbiome analyses, stable-isotope tracing, and ultimately clinical studies will be required to address these limitations.

Taken together, the present study shows that cycloartane TTGs of *Cimicifuga racemosa* are extensively transformed by human fecal microbiota, most likely through initial deglycosylation followed by further metabolism of the resulting aglycones. At the same time, exposure to TTGs or Ze 450 did not substantially alter overall microbial community composition or SCFA production under the investigated conditions. The marked donor-dependent differences in aglycone recovery demonstrate considerable interindividual variation in microbial metabolic capacity that cannot be explained solely by the abundance of dominant bacterial families. These findings provide a mechanistic basis for investigating whether microbiome-dependent differences influence the pharmacokinetics or clinical effects of Ze 450. Larger donor cohorts, functional microbiome analyses, time-resolved metabolomics, and ultimately clinical studies will be required to validate the proposed metabolic phenotypes and determine their clinical relevance.

## 4. Materials and Methods

### 4.1. Test Compounds

To investigate the intestinal microbial metabolism of *Cimicifuga racemosa*, anaerobic batch fermentation experiments were performed using an experimental setup adapted from Chauveau et al. [34]. The *Cimicifuga racemosa* extract Ze 450 (Max Zeller Söhne AG, Romanshorn, Switzerland; batch number 222440) and four pure triterpene glycosides (TTGs)—actein (TTG-1), 23-epi-26-deoxyactein (formerly 27-deoxyactein; TTG-2), cimigenol-3-O-β-D-xyloside (TTG-3), and cimigenol-3-O-α-L-arabinoside (TTG-4)—were investigated. The TTGs were obtained as phyproof^®^ primary reference substances (purity > 98%) from PhytoLab GmbH & Co. KG (Vestenbergsgreuth, Germany). Stock solutions of the TTGs (15 mg/mL) and Ze 450 (150 mg/mL) were prepared in DMSO and added to 2 mL anaerobic fecal fermentation incubations to achieve final concentrations of 30 µg/mL for each TTG and 300 µg/mL for Ze 450. The TTG concentration was selected based on the concentration used by Chauveau et al. [34] for single compounds in short-term gut microbiota fermentation experiments. Ze 450 was tested at 300 µg/mL, corresponding to approximately 30 µg/mL total triterpene glycosides based on a total TTG content of approximately 10%, expressed as 23-epi-26-deoxyactein. Thus, the total TTG concentration provided by Ze 450 was comparable in magnitude to the 30 µg/mL concentration used for the individual TTGs. The final DMSO concentration in all incubations was 0.2% (*v*/*v*). TTG degradation and quantitative aglycone formation were analyzed by UHPLC-qTOF-MS. Changes in microbial community composition were assessed by 16S rRNA gene sequencing using Illumina MiSeq, and short-chain fatty acids (SCFAs) were quantified by LC-RI.

### 4.2. Simulation of Gastrointestinal Conditions: Infogest

Actein, 23-epi-26-deoxyactein, cimigenol-3-β-D-xyloside, cimigenol-3-α-L-arabinoside, and the *Cimicifuga racemosa* extract Ze 450 were dissolved in methanol (1 mg/mL) and diluted in water to working concentrations of 20 and 2 µg/mL. The higher concentration was chosen to approximate the concentration range used in the ex vivo fecal fermentation experiments (30 µg/mL), whereas the lower concentration represented a ten-fold dilution. Simulated static in vitro gastrointestinal digestion was performed in duplicate in 96-well deep-well plates according to the standardized INFOGEST protocol [32]. Simulated salivary, gastric, and intestinal fluids were prepared as described in the protocol, including the recommended electrolytes, digestive enzymes, and bile salts, except that sodium bicarbonate was replaced with sodium chloride.

Briefly, samples underwent sequential oral (2 min, pH 7, α-amylase), gastric (2 h, pH 3, rabbit gastric extract providing gastric lipase and pepsin), and intestinal (2 h, pH 7, pancreatin and bile extract) digestion at 37 °C under agitation. At the end of each digestion phase, enzymes were inactivated by heating at 95 °C for 5 min, and samples were stored at −80 °C until analysis. To assess non-enzymatic degradation, compounds were incubated under identical conditions in water without digestive fluids. For the stability assessment reported in Section 2.1 and Figure S1, analytical evaluation focused on samples collected after the oral and gastric phases.

### 4.3. Growth Media and Anaerobic Procedures

The cultivation media for ex vivo cultivation of fecal microbiota was the basal version of YCFA (referred to as bYCFA-6C+muc), which was previously described in detail, and validated for maximizing compositional similarity to feces [45]. Compared to the original YCFA formulation, bYCFA contained tenfold less N-sources and a complex C-source mixture (6C+muc) with 0.45 g/L starch (from potato; Sigma-Aldrich, Buchs, Switzerland), 0.45 g/L pectin (from citrus peel; Sigma-Aldrich), 0.45 g/L xylan (from oat spelt; Angene, London, UK), 0.24 g/L arabinogalactan (from larch wood; Sigma-Aldrich), 0.24 g/L guar (Sigma-Aldrich), 1.14 g/L inulin (Beneo, Mannheim, Germany; Sigma-Aldrich) and 0.3 g/L mucin (from porcine stomach type II; Sigma-Aldrich).

The final growth medium bYCFA-6C+muc was prepared by mixing three parts of the concentrated medium without C-sources (1.33-fold concentrated bYCFA) and one part of separately prepared C-source solution (4-fold concentrated 6C+muc). For the medium preparation, all components, except L-cysteine and sodium bicarbonate, were dissolved in 75% of the intended volume and pH was adjusted to 7. After boiling for 10 min and cooling down to 60 °C, under continuous stirring and CO_2_-flushing, the remaining ingredients were added. After 10 min, the medium was autoclaved in DURAN^®^ Pressure flasks (DWK Life Sciences GmbH, Wertheim, Germany) or Hungate tubes (Belleco Glass, Vineland, NJ, USA). The same procedure was followed for the C-source solutions, except that N_2_ was used to flush the solutions.

### 4.4. Ex Vivo Cultivation and Gut Microbiota Responses to Ze 450

Ex vivo cultivation of fecal microbiota was performed according to a previously optimized protocol [45]. Fresh fecal samples were provided by 8 healthy human female adults (21–37 years old). Donors were excluded if they had taken antibiotics in the past 6 months. Samples were collected anonymously; therefore, additional donor metadata, including menstrual-cycle phase, hormonal contraception, BMI, habitual diet, and concomitant medication, were not available. The ethics committee of ETH Zurich exempted this study from review because the sample collection procedure was processed in an anonymized, voluntary and non-interventional manner. Self-collected samples were provided in plastic containers containing an Oxoid™ AnaeroGen™ pouch (Thermo Fisher Diagnostics AG, Pratteln, Switzerland) to guarantee anaerobiosis. Samples were transported into the anaerobic chamber within three hours for immediate processing. Fecal sample dilutions (10^−4^ in anaerobic phosphate buffer) were inoculated (1% *v*/*v*, technical triplicates) into 96-deep-well plates containing bYCFA-6C+muc medium (pH 6.5) supplemented with individual triterpene glycosides (30 µg/mL each), Ze 450 (300 µg/mL), or vehicle control (0.2% DMSO, *v*/*v*). Cultures were incubated anaerobically for 48 h at 37 °C. For subsequent analysis, the technical replicates were pooled by transferring 0.5 mL from culture of each technical replicate into anew plate [45]. From the pooled samples, supernatants were collected and cell pellets by centrifugation (5′500 rpm, 20 min, 4 °C) for metabolic and microbial analyses, respectively.

### 4.5. Organic Acids Quantification

Organic acid production (i.e., acetate, propionate, butyrate, formate, lactate and succinate) in sterile-filtered culture supernatants (collected by centrifugation, 5500 rpm, 20 min, 4 °C) was measured using a Hitachi LaChrom HPLC (Merck, Dietikon, Switzerland), equipped with a pre-column SecurityGuard Cartridges Carbo-H (4 × 3.0 mm; Phenomenex Helvetia GmbH, Basel, Switzerland) and a Rezex ROA-Organic Acid H + column (300 × 7.8 mm; Phenomenex Helvetia GmbH), and a refractive index detector [46]. All concentrations were corrected with blank values of the un-inoculated medium to estimate the net production of individual metabolites. Total metabolite production was estimated by summing all organic acid concentrations (i.e., acetate, propionate, butyrate, formate, lactate and succinate). Metabolite fractions were calculated by dividing the concentration of individual metabolites by total metabolites.

### 4.6. DNA Extraction and Community Composition Analysis Using 16S rRNA Gene Amplicon Sequencing

The effect of TTGs and Ze 450 on the community composition was tested using 16S rRNA gene sequencing. Cell pellets underwent pre-processing utilizing the Maxwell^®^ HT 96 gDNA Blood Isolation Kit (Promega AG, Dübendorf, Switzerland) and a FastPrep homogenizer (FastPrep-24TM; MP Biomedicals, Illkirch-Graffenstaden, France) according to the manufacturer’s instructions. Subsequently, DNA extraction was carried out using the KingFisher Flex instrument (Thermo Fisher Scientific, Waltham, MA, USA). Amplification and barcoding of the V4 hypervariable region of the 16S rRNA gene were accomplished through polymerase chain reaction (PCR) with the barcoded primer 515F (5′-GTGCCAGCMGCCGCGGTAA-3′) and 806R (5′-GGACTACHVGGGTWTCTAAT-3′) (Integrated DNA Technologies, Leuven, Belgium). The combined library was sequenced using the amplifying primers 515F and 806R and the Illumina MiSeq platform at the Genetic Diversity Centre (ETH Zurich, Switzerland).

### 4.7. Preparation and Purity Assessment of Aglycone Reference Standards

Since a commercial aglycone reference standard was not available, 27-deoxyacetylacteol was prepared in-house by enzymatic hydrolysis of 23-epi-26-deoxyactein. For hydrolysis, 5 mg of 23-epi-26-deoxyactein was incubated with 1000 mg of cellulase from Trichoderma viride (VWR, Radnor, PA, USA) in 200 mL of phosphate-buffered saline (PBS; Corning, NY, USA). The reaction mixture was incubated at 37 °C overnight under agitation at 250 rpm. After incubation, the reaction was terminated by liquid–liquid extraction. The aglycone fraction was extracted three times with hexane (VWR, Radnor, PA, USA). The combined organic phases were concentrated by rotary evaporation and subsequently dried under a stream of nitrogen. The resulting residue was used as an in-house reference standard for 27-deoxyacetylacteol. The identity of the generated aglycone was assigned by liquid chromatography–mass spectrometry based on chromatographic retention behavior and the expected monoisotopic sodium adduct. The precursor compound 23-epi-26-deoxyactein was detected at a retention time of 19.7 min with an expected [M + Na]^+^ ion at *m*/*z* 683.3771. The enzymatically generated aglycone, 27-deoxyacetylacteol, was detected at a retention time of 28.0 min with an expected [M + Na]^+^ ion at *m*/*z* 551.3349.

Chromatographic purity was assessed by UPLC-PDA-MS on a Waters UPLC H-Class system equipped with a quaternary pump, photodiode array detector (PDA) detector, and quadrupole detector (QDA) (Milford, MA, USA). Separation was achieved on a Zorbax Eclipse XDB C18 column (250 × 4.6 mm, 5 µm; Agilent Santa Clara, CA, USA) maintained at 25 °C. The autosampler temperature was 25 °C, the injection volume was 10 µL, and the flow rate was 1.0 mL/min. Mobile phase (A) consisted of 0.1% formic acid in water and mobile phase (B) of 0.1% formic acid in methanol/acetonitrile (1:1, *v*/*v*). The gradient was performed as follows: 0.0 min, 50% B; 40.0 min, 80% B; 41.0 min, 95% B; 49.0 min, 95% B; 50.0 min, 50% B; 60.0 min, 50% B. Detection was performed in positive ionization full-scan mode with a cone voltage of 20 V, a capillary voltage of 1.2 kV and a probe temperature of 450 °C.

Purity was estimated using a total area content approach based on integration of all detectable chromatographic peaks in the full-scan chromatogram. The purity of the isolated aglycone 27-deoxyacetylacteol was 97.15%.

### 4.8. Extraction and UHPLC-MS Analysis of Fecal Incubation Samples

For metabolite analysis, 0.4 mL aliquots of fecal incubation samples were extracted in 2 mL tubes. To recover TTGs, samples were extracted three times with 0.4 mL water-saturated ethyl acetate. To ensure full recovery of the corresponding aglycones, the remaining aqueous phase was subsequently extracted two times with 0.4 mL hexane. During each extraction step, samples were vortex-mixed for 30 s and allowed to separate for 5 min. The organic phases were combined, evaporated to dryness under a stream of nitrogen, and reconstituted in 0.5 mL methanol. Samples were centrifuged prior to analysis and stored at −20 °C until measurement. α-Hederin (purity > 95%) purchased from Phytolab (Vestenbergsgreuth, Germany) was used as an internal standard to compensate for variability in sample matrix and instrumental response. It was added to all samples after extraction at a final concentration of 250 ng/mL.

Qualitative and quantitative analysis of TTGs and aglycones were performed using LC-PDA-HRMS on a Waters UPLC H-Class system with a quaternary pump, PDA detector, and coupled to a Waters SYNAPT G2-S Q-TOF (Waters, Milford, MA, USA) with an ESI source operated in positive ion mode within a scan range of 100–900 Da. Chromatographic separation was carried out under the same LC conditions as described above for aglycone purity assessment. An adjustable flow splitter (ASI, El Sobrante, CA, USA) was used to direct 25% of the LC flow to the mass spectrometer. TTGs and their corresponding aglycones were monitored in full-scan mode based on their monoisotopic sodium-adduct ions [M + Na]^+^. The monitored TTG ions were *m*/*z* 683.3766 for 23-epi-26-deoxyactein, *m*/*z* 699.3715 and 467.3128 for actein, and *m*/*z* 643.3817 for both cimigenol-3-β-D-xyloside and cimigenol-3-α-L-arabinoside. The corresponding aglycone ions were *m*/*z* 551.3343 for 27-deoxyacetylacteol, *m*/*z* 567.3292 for acetylacteol, and *m*/*z* 511.3394 for cimigenol. Additionally, a characteristic ion at *m*/*z* 467.3128 was detected for actein and acetylacteol, consistent with the presence of a sodiated fragment ion [C_28_H_44_O_4_ + Na]^+^. Data acquisition and processing were performed using MassLynx and TargetLynx software (Waters, Milford, MA, USA).

Calibration curves for TTG’s were prepared at 30–500 ng/mL and at 40–700 ng/mL for 23-epi-26-deoxyactein, using five concentration levels each. Quantification was based on analyte-to-internal-standard peak area ratios using α-hederin as the internal standard. Linear regression was applied, and calibration acceptance required a coefficient of determination (R^2^) > 0.99. Although calibration solutions were established for several TTGs and aglycones, quantitative evaluation in the present study focused on the conversion of 23-epi-26-deoxyactein to 27-deoxyacetylacteol. 27-deoxyacetylacteol was quantified using the isolated in-house aglycone standard, whereas the remaining TTGs and aglycones were assessed qualitatively.

### 4.9. Bioinformatics and Statistical Analysis

Raw sequencing reads were processed using the metabaRpipe R package (v0.9) [47]. Sequence inference was performed using DADA2 (v1.14.1), with reads truncated at positions 140 and 160 and maximum expected error rates set to 2. Amplicon sequence variants (ASVs) were taxonomically assigned using the SILVA database (v138.1) [48] and subsequently analyzed using the phyloseq package (v1.40.0) [49] in R (v4.2.0). Low-abundance reads were filtered using a per-sample abundance threshold of 0.1% [50].

Alpha- and beta-diversity analyses were conducted on rarefied data, retaining 2374 reads per sample. Alpha-diversity was assessed using the Shannon diversity index and observed amplicon sequence variants (ASVs), and differences between experimental conditions were tested using paired Wilcoxon signed-rank tests. Beta-diversity was assessed using Bray–Curtis dissimilarity. For each donor, Bray–Curtis distances between treatment and corresponding DMSO control samples were calculated, and differences among treatment conditions were evaluated using paired Wilcoxon signed-rank tests with Benjamini–Hochberg correction for multiple testing. Compositional taxa bar plots were generated using the comp_barplot () function of the microViz package (v0.10.8) [51].

Differential abundance was assessed using ALDEx2 [52]. Unrarefied read data were transformed to centered log-ratios (clr) using 128 Dirichlet Monte Carlo instances and the aldex.clr function from the mia package (v1.5.17) [53]. Differentially abundant taxa were identified using Welch’s *t*-test, and *p* values were adjusted for multiple comparisons using the Bonferroni correction [45]. Genus-level differences between treatment conditions and the corresponding DMSO controls were assessed using the Wilcoxon rank-sum test. Adjusted *p* values < 0.05 were considered statistically significant. Given the limited number of donors, all statistical analyses were considered exploratory.

## Figures and Tables

**Figure 1:**
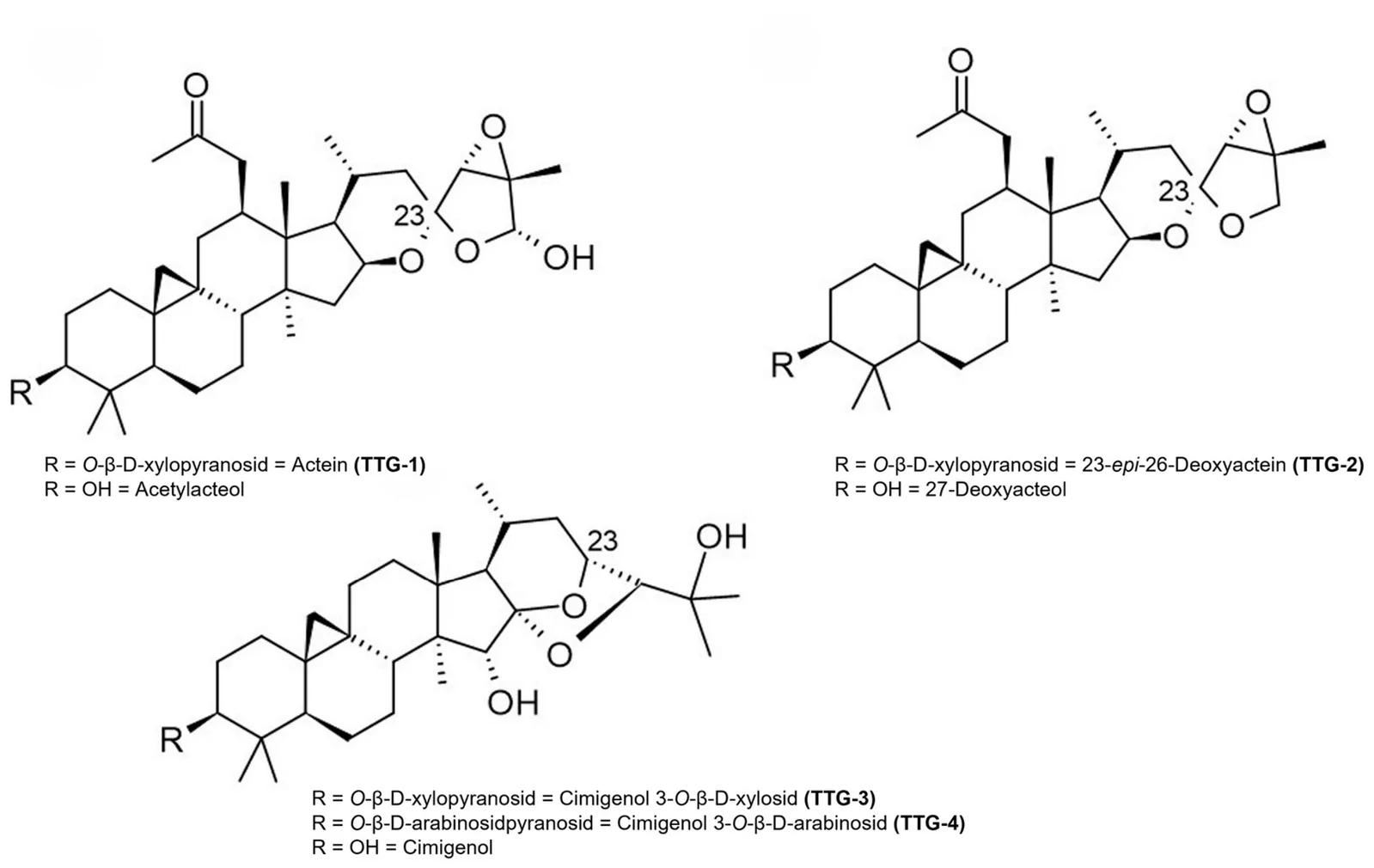
Chemical structures of the selected Cimicifuga racemosa triterpene glycosides (TTGs) investigated in this study and their corresponding aglycones.

**Figure 2:**
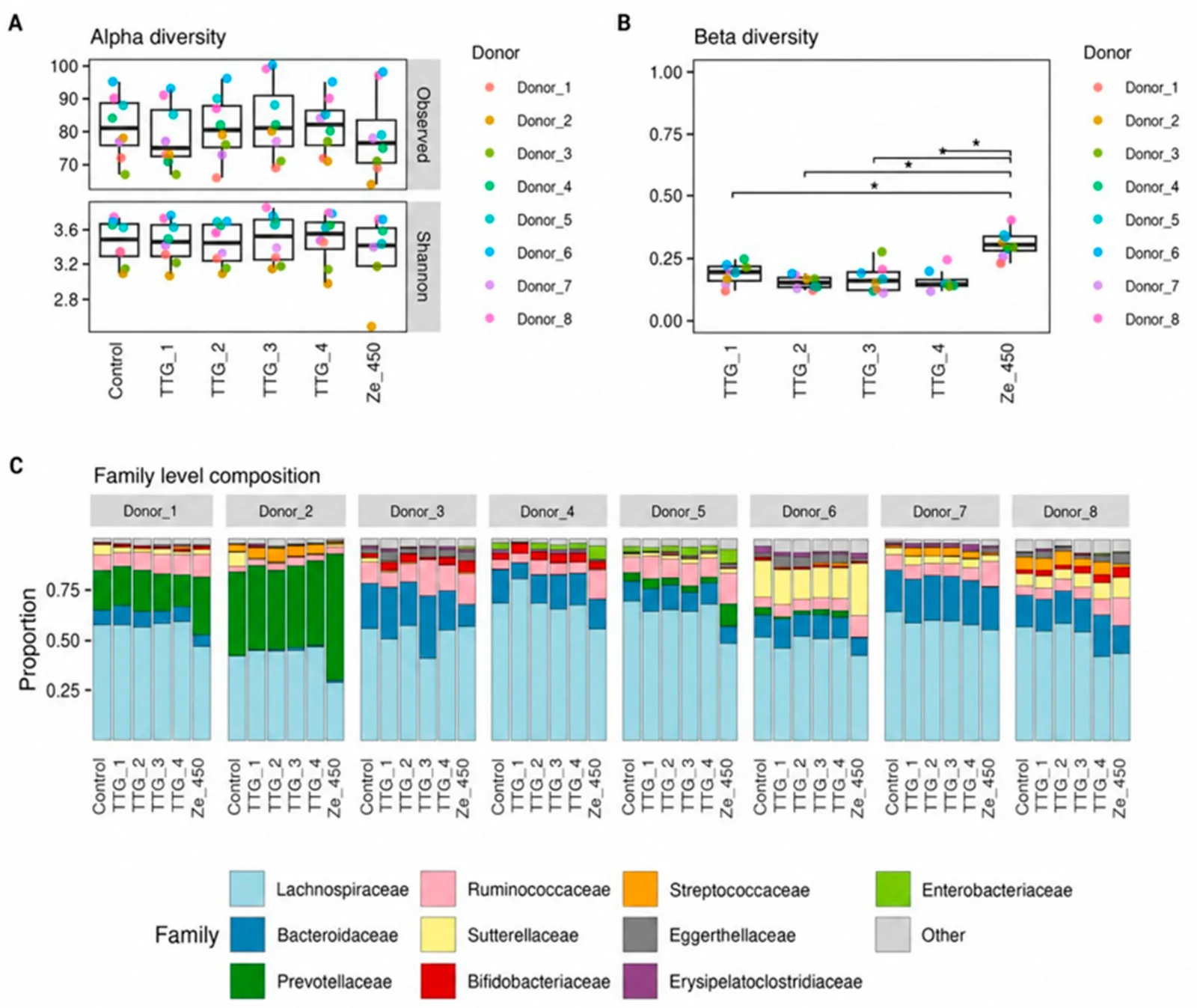
Effect of *Cimicifuga racemosa* triterpene glycosides on microbial community composition based on 16S rRNA gene sequencing. (**A**) Alpha diversity of fecal microbial communities from eight in-dividual donors (1–8) following treatment with DMSO (vehicle control), four triterpene glycosides (TTG-1 to TTG-4; 30 µg/mL each), or Ze 450 (*C. racemosa* extract; 300 µg/mL). The upper panel shows observed amplicon sequence variants (ASVs; richness), and the lower panel shows the Shannon index (richness and evenness). Statistical analysis was performed using paired Wilcoxon tests with Benjamini–Hochberg (BH) correction, with DMSO as the reference group. No significant differences were observed. (**B**) Beta diversity analysis based on Bray–Curtis distances between DMSO controls and treatment conditions (TTG-1 to TTG-4 and Ze 450). A Bray–Curtis distance of 0 indicates identical community composition, whereas a value of 1 indicates complete dissimilarity. Colors indicate individual donors. Statistical analysis was per-formed using paired Wilcoxon tests with Benjamini–Hochberg (BH) correction. Significant differences were detected between Ze 450 and all other treatment conditions (*p* = 0.02 for all comparisons). (**C**) Relative taxonomic abundances of microbial communities (family level) across treatment conditions. ASV, amplicon sequence variant. TTG-1, actein; TTG-2, 23-epi-26-deoxyactein; TTG-3, cimigenol-3-β-D-xyloside; TTG-4, cimigenol-3-α-L-arabinoside. Asterisks (*) indicate statistically significant differences between Ze 450 and the respective TTG treatment conditions (*p* = 0.02 for all comparisons; paired Wilcoxon signed-rank test with Benjamini–Hochberg correction).

**Figure 3:**
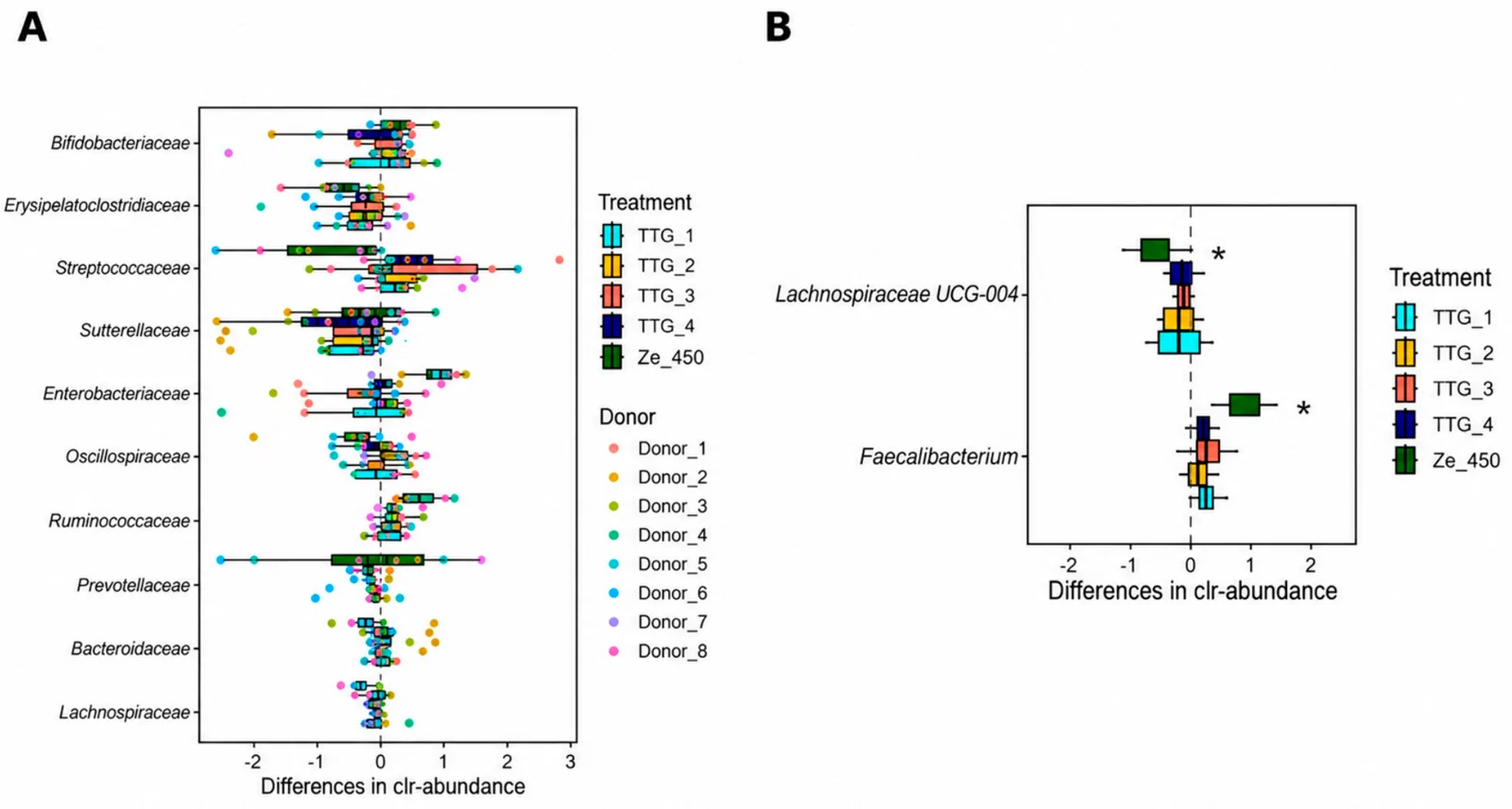
Differential abundance analysis reveals only minor effects of TTGs and Ze 450 on specific intestinal taxa. Fecal samples from eight individual female donors (18) were treated with four triterpene glycosides (TTG1 to TTG-4; 30 µg/mL each), Ze 450 (*Cimicifuga racemosa* extract; 300 µg/mL), or DMSO (vehicle control). Differential abundance analyses were performed relative to the DMSO control. Colors indicate individual donors. (**A**) Differential abundance analysis at the family level. The 10 most abundant bacterial families are shown. Statistical analysis was performed using ALDEx2; no significant differences were detected between treatment and control conditions. (**B**) Differential abundance analysis at the genus level. Statistical significance was assessed using the Wilcoxon rank-sum (Mann–Whitney) test. Two genera showed significant differences relative to the DMSO control. TTG1, actein; TTG2, 23-epi-26-deoxyactein; TTG3, cimigenol-3-β-D-xyloside; TTG4, cimigenol-3-α-L-arabinoside. Asterisks indicate statistically significant differences compared with the DMSO control (Lachnospiraceae UCG-004, *p* = 0.047; Faecalibacterium, *p* = 0.033; Wilcoxon rank-sum test).

**Figure 4:**
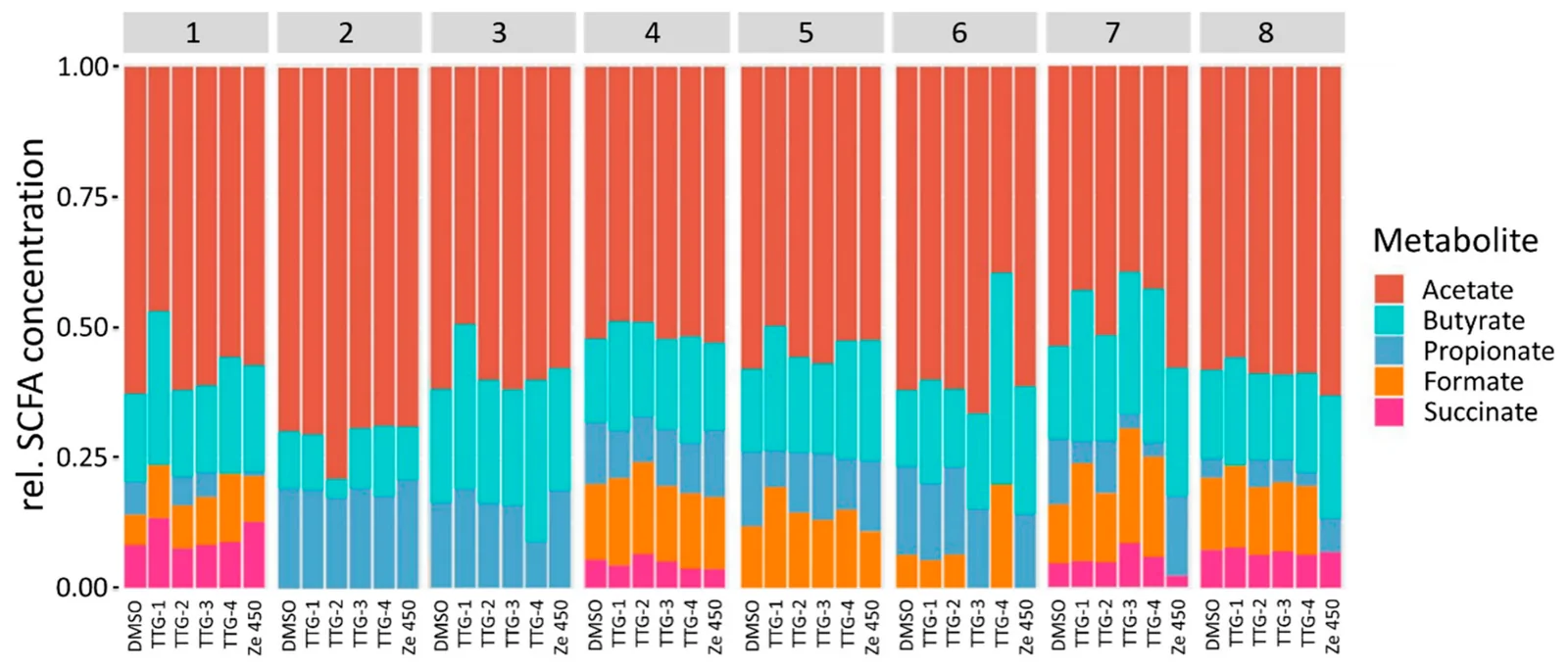
Interindividual variability of microbial metabolic profile is only minimally affected by TTGs and Ze 450. Bars show relative concentrations of short-chain fatty acids (SCFAs) in fecal samples from eight individual female donors (1–8) following treatment with four triterpene glycosides (TTG-1 to TTG-4; 30 µg/mL each), Ze 450 (*Cimicifuga racemosa* extract; 300 µg/mL), or DMSO (vehicle control). The concentrations of acetate, butyrate, propionate, formate, and succinate were quantified by High Performance Liquid Chromatography with refractive index detection (HPLC-RI).

**Figure 5:**
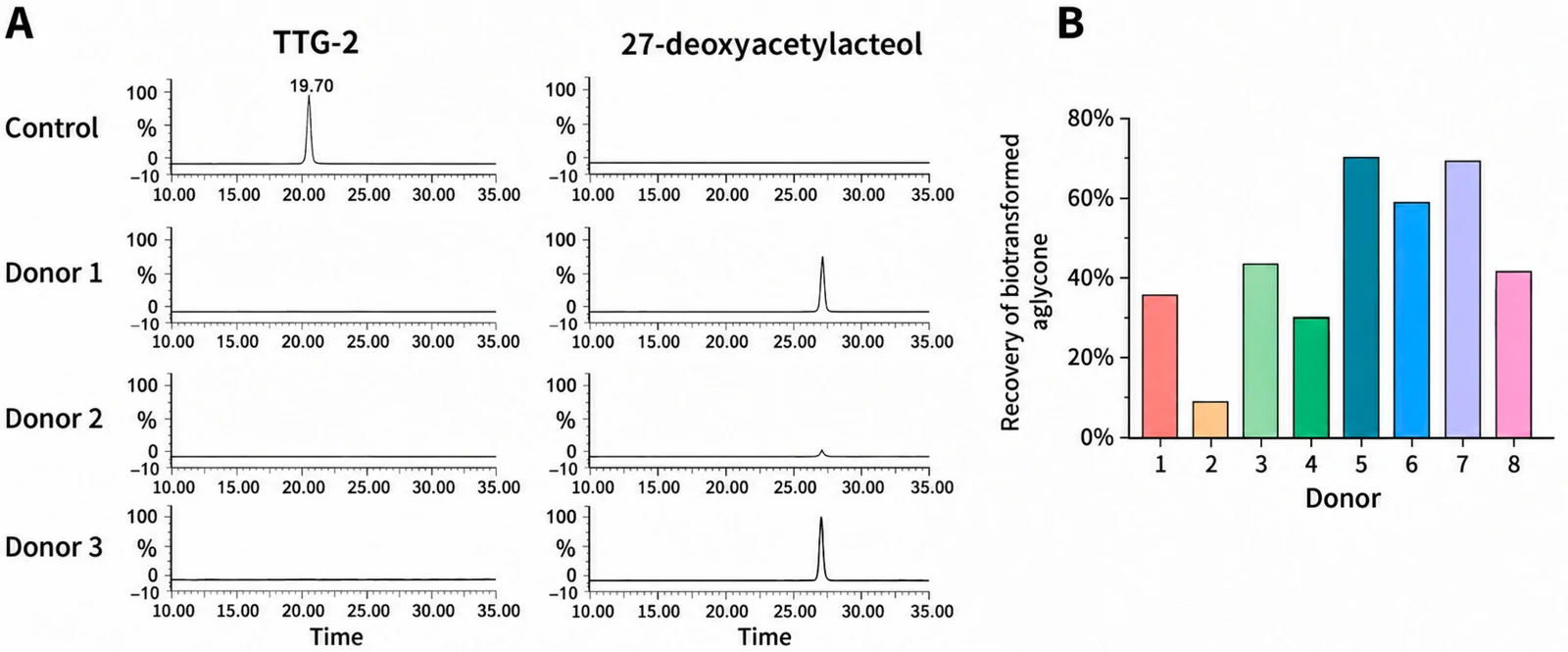
Aglycone recovery appears to be donor- and thus microbiome-specific. Fecal samples from eight female donors (1–8) were analyzed by UHPLC-qTOF-MS after 48 h anaerobic incubation with 23-epi-26-deoxyactein (TTG-2, 30 µg/mL). DMSO was used as vehicle control. (**A**) Qualitative analysis of 23-epi-26-deoxyactein degradation. Representative chromatograms of donors 1, 2 and 3 show absence of the glycoside (left panel) and presence of the aglycones (right panel) upon incubation. (**B**) Quantitative analysis of 27-deoxyacteylacteol (aglycone) recovery. Bars show mean technical duplicates. Colors indicate respective donors.

## Data Availability

Data are available on request from the authors.

## Supplementary Materials

The following supporting information can be downloaded at: https://www.mdpi.com/article/10.3390/molecules31213928/s1. Figure S1: Stability of TTG-2 (23-epi-26-deoxyactein) in model oral and gastric phases to ensure bioavailability in the intestine. TTG-2 was incubated for 2 min at pH 7.0 in simulated salivary fluid with CaCl2 and Amylase and subsequently for 2 h at pH 3.0 in simulated gastric fluid with CaCl2, pepsin and gastric lipase. Samples were analyzed LC-HRMS as described in the Material & Method section. TTG-2 recovery [%] has been calculated relative to control samples. Bars show mean of technical duplicates; Figure S2: Microbiota-mediated degradation of Actein (TTG-1) and formation of the corresponding aglycone (Acetylacteol). Representative UHPLC-qTOF-MS chromatograms obtained after 48 h of anaerobic incubation with human fecal microbiota show depletion of actein (TTG-1) and appearance of the corresponding aglycone. As both actein and its aglycone are detected at the same mass by LC-HRMS, the dashed lines indicate the retention time of actein (left) and its aglycone (right) DMSO served as vehicle control; Figure S3: Microbiota-mediated degradation of Cimigenol 3-O-β-D-xylosid (TTG-3) and formation of the corresponding aglycone (Cimigenol). Representative UHPLC-qTOF-MS chromatograms obtained after 48 h of anaerobic incubation with human fecal microbiota show depletion of cimigenol-3-O-β-D-xylopyranosid (TTG-3) and appearance of the corresponding aglycone. DMSO served as vehicle control; Figure S4: Microbiota-mediated degradation of Cimigenol 3-O-β-D-arabinosid (TTG-4) and formation of the corresponding aglycone (Cimigenol). Representative UHPLC-qTOF-MS chromatograms obtained after 48 h of anaerobic incubation with human fecal microbiota show depletion of cimigenol-3-O-α-L-arabinopyranosid (TTG-4) and appearance of the corresponding aglycone. DMSO served as vehicle control.

## Abbreviations

The following abbreviations are used in this manuscript:

ALDEx2	Analysis of Differential Abundance Taking Sample Variation Into Account
AMPK	AMP-activated protein kinase
ASV	Amplicon sequence variant
BH	Benjamini–Hochberg
CLR	Centered log-ratio
DMSO	Dimethyl sulfoxide
DNA	Deoxyribonucleic acid
ESI	Electrospray ionization
HPLC	High-performance liquid chromatography
HPLC-RI	High-performance liquid chromatography with refractive index detection
HRMS	High-resolution mass spectrometry
LC	Liquid chromatography
LC-HRMS	Liquid chromatography-high-resolution mass spectrometry
LC-PDA-HRMS	Liquid chromatography-photodiode array-high-resolution mass spectrometry
PBS	Phosphate-buffered saline
PCR	Polymerase chain reaction
PDA	Photodiode array
QDA	Quadrupole detector
Q-TOF	Quadrupole time-of-flight
rRNA	Ribosomal RNA
SCFA	Short-chain fatty acid
TTG	Triterpene glycoside
UHPLC	Ultra-high-performance liquid chromatography
UHPLC-qTOF-MS	Ultra-high-performance liquid chromatography-quadrupole time-of-flight mass spectrometry
UPLC	Ultra-performance liquid chromatography
YCFA	Yeast extract-casitone-fatty acid medium
