# Supplementary material for: Gastrointestinal Stability and Microbiota-Mediated Deglycosylation of Triterpene Glycosides from Cimicifuga racemosa Extract Ze 450
Source: Molecules. 2026 Nov 1;31(21):3928. doi: 10.3390/molecules31213928 (PMC13765399; doi:10.3390/molecules31213928)
Supplement: Supplementary file 1 [file molecules-31-03928-s001.zip › molecules-4621728-supplementary.pdf]

Supplementary Material

Microbiota-mediated Biotransformation of Triterpene Glycosides from *Cimicifuga racemosa* extract Ze 450

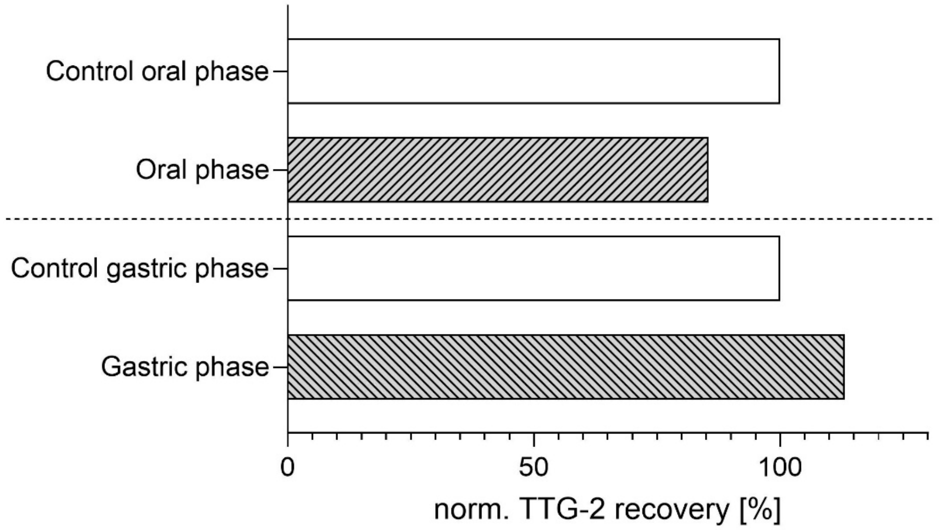

Figure S1: Stability of TTG-2 (23-epi-26-deoxyactein) in model oral and gastric phases to ensure bioavailability in the intestine. TTG-2 was incubated for 2 min at pH 7.0 in simulated salivary fluid with CaCl2 and Amylase and subsequently for 2 h at pH 3.0 in simulated gastric fluid with CaCl2, pepsin and gastric lipase. Samples were analyzed LC-HRMS as described in the Material & Method section. TTG-2 recovery [%] has been calculated relative to control samples. Bars show mean of technical duplicates.

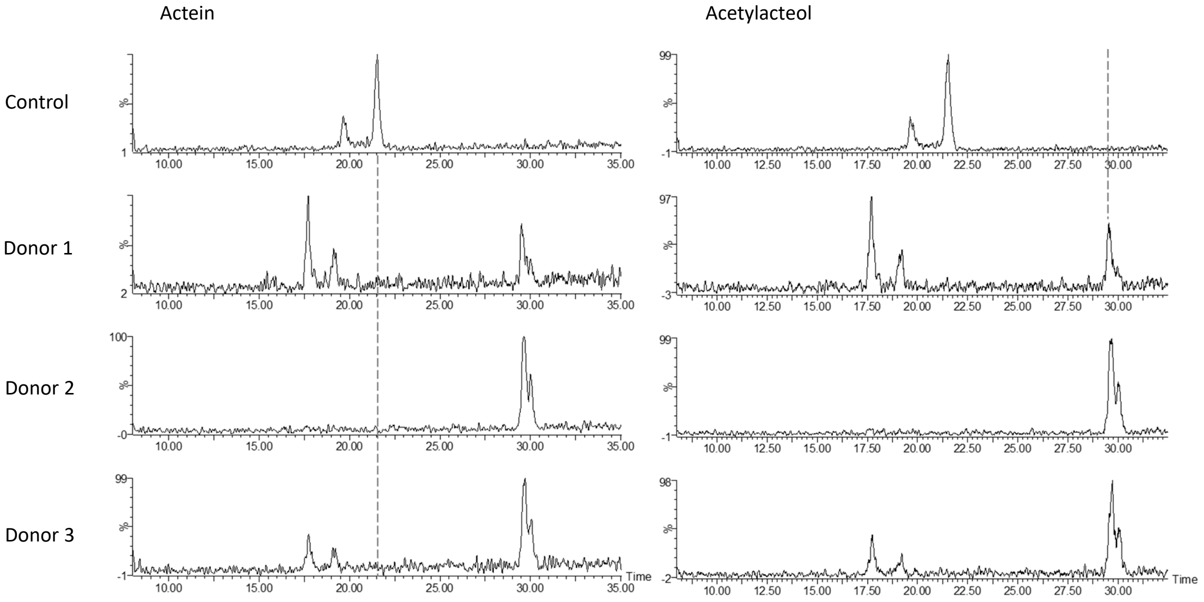

Figure S2: Microbiota-mediated degradation of Actein (TTG-1) and formation of the corresponding aglycone (Acetylacteol). Representative UHPLC-qTOF-MS chromatograms obtained after 48 h of anaerobic incubation with human fecal microbiota show depletion of actein (TTG-1) and appearance of the corresponding aglycone. As both actein and its aglycone are detected at the same mass by LC-HRMS, the dashed lines indicate the retention time of actein (left) and its aglycone (right) DMSO served as vehicle control.

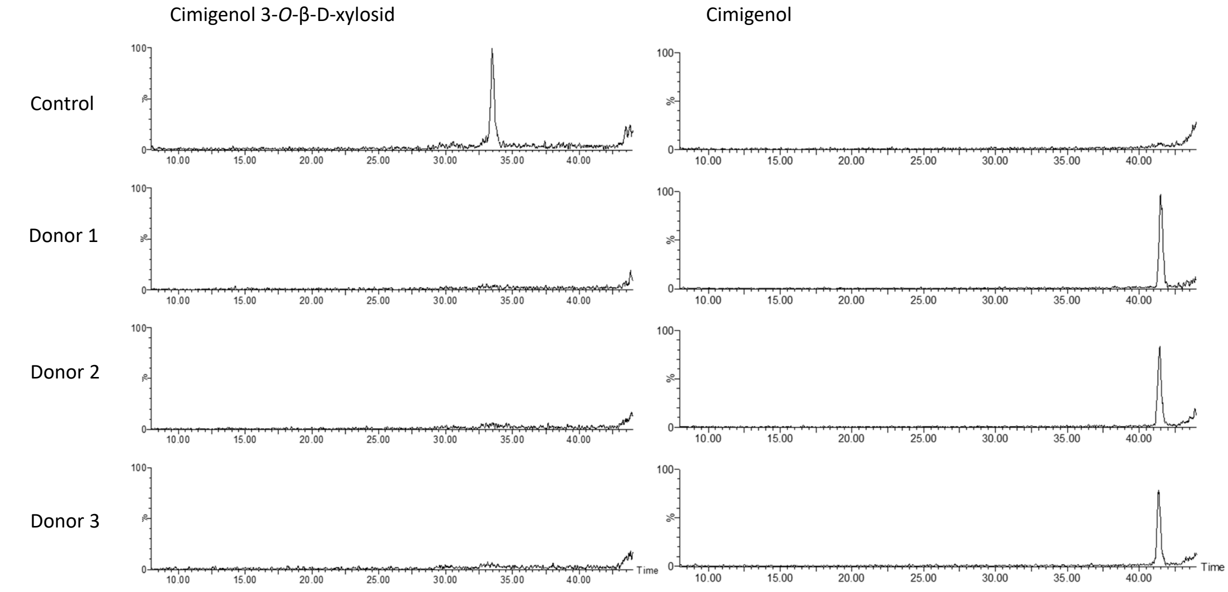

Figure S3: Microbiota-mediated degradation of Cimigenol 3-O-β-D-xylosid (TTG-3) and formation of the corresponding aglycone (Cimigenol). Representative UHPLC-qTOF-MS chromatograms obtained after 48 h of anaerobic incubation with human fecal microbiota show depletion of cimigenol-3-*O*-β-D-xylopyranosid (TTG-3) and appearance of the corresponding aglycone. DMSO served as vehicle control.

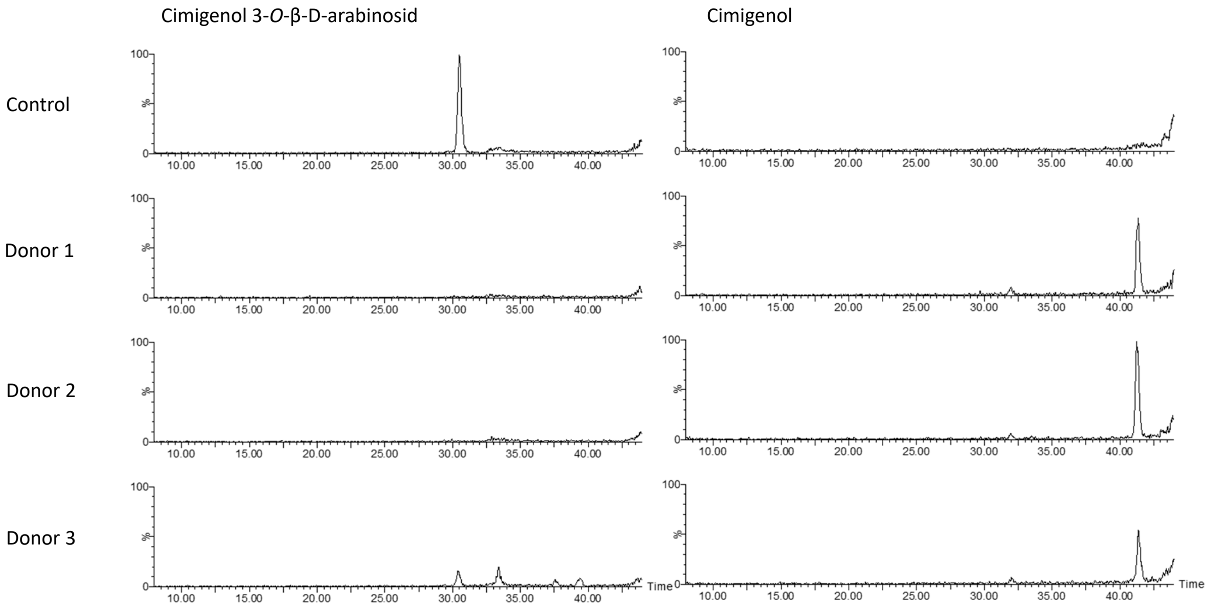

Figure S4: Microbiota-mediated degradation of Cimigenol 3-*O*-β-D-arabinosid (TTG-4) and formation of the corresponding aglycone (Cimigenol). Representative UHPLC-qTOF-MS chromatograms obtained after 48 h of anaerobic incubation with human fecal microbiota show depletion of cimigenol-3-*O*-α-L-arabinopyranosid (TTG-4) and appearance of the corresponding aglycone. DMSO served as vehicle control.
